# IL15.3, a synthetic antimicrobial peptide, attenuates LPS-induced inflammatory responses in RAW 264.7 macrophages via transcriptional and proteomic modulation

**DOI:** 10.1371/journal.pone.0361031

**Published:** 2026-10-22

**Authors:** Chanapat Boonmark, Sittiruk Roytrakul, Fasawanya Saengyotha, Anupong Tankrathok, Anuwat Wanthong, Jureerut Daduang, Sakda Daduang, Arunrat Chaveerach, Nisachon Jangpromma

**Affiliations:** 1 Protein and Bio-Innovation Research Center for Sustainable Health (ProBISH), Faculty of Science, Khon Kaen University, Khon Kaen, Thailand; 2 National Center for Genetic Engineering and Biotechnology, National Science and Technology Development Agency, Pathumthani, Thailand; 3 Faculty of Agricultural Technology, Department of Biotechnology, Kalasin University, Kalasin, Thailand; 4 Department of Biology, Faculty of Science, Mahasarakham University, Mahasarakham, Thailand; 5 Department of Clinical Chemistry, Faculty of Associated Medical Sciences, Khon Kaen University, Khon Kaen, Thailand; 6 Department of Pharmacognosy and Toxicology, Faculty of Pharmaceutical Sciences, Khon Kaen University, Khon Kaen, Thailand; 7 Department of Biology, Faculty of Science, Khon Kaen University, Khon Kaen, Thailand; 8 Department of Biochemistry, Faculty of Science, Khon Kaen University, Khon Kaen, Thailand; Pacific Northwest National Laboratory, UNITED STATES OF AMERICA

## Abstract

Inflammation underlies numerous pathological conditions, driving the need for safe and effective anti-inflammatory agents. Anti-inflammatory peptides (AIPs) are promising therapeutics due to their specificity, efficacy, and safety. This study evaluated the cationic antimicrobial peptide IL15.3 for anti-inflammatory activity and cytocompatibility *in vitro*. IL15.3 demonstrated low cytotoxicity in MTT assays using RAW 264.7 macrophages, normal human dermal fibroblasts (NHDF), and HaCaT keratinocytes. In LPS-stimulated RAW 264.7 cells, IL15.3 significantly reduced nitric oxide (NO) production and reactive oxygen species (ROS) in a dose-dependent manner without affecting cell viability. qRT-PCR analysis revealed significant suppression of *iNOS*, *COX-2*, *TNF-α*, and *IL-6* mRNA expression, alongside downregulation of *NF-κB*, *JNK*, *STAT1*, and *STAT3*, and a reciprocal shift in *Keap1*/*Nrf2* expression suggestive of possible antioxidant pathway activation. Label-free proteomic profiling identified 5,191 proteins, revealing a multi-modal mechanism of action. IL15.3 treatment exclusively induced 4 anti-inflammatory proteins (ISG15, PEX14, TNIP3, PR) and upregulated negative regulators NRG1, DAB2IP, IL-18BP and SOCS5. Critically, IL15.3 suppressed key pro-inflammatory mediators including IKKβ, JAK1, JAK3, JNK, and IL-1RAcP, demonstrating comprehensive modulation of NF-κB, JAK/STAT, and MAPK pathways. *In silico* molecular docking further predicted potential interactions of IL15.3 with iNOS and the Keap1 Kelch domain, offering preliminary structural insight consistent with its putative dual anti-inflammatory and antioxidant activity. Together, these findings indicate that IL15.3 exhibits potent anti-inflammatory and antioxidant activity through multiple mechanisms in LPS-stimulated macrophages at transcriptional and proteomic levels. The coordinated upregulation of negative regulators and suppression of central inflammatory mediators collectively suggest that IL15.3 may represent a promising therapeutic candidate for inflammatory disorders.

## 1. Introduction

Inflammation is a fundamental, evolutionarily conserved host response critical for restoring tissue integrity after infection or injury [1]. This intricate process involves the recruitment of immune cells and the activation of coordinated transcriptional programs, including the expression of cytokines, chemokines, and inducible enzymes such as inducible nitric oxide synthase (iNOS) and cyclooxygenase-2 (COX-2) [1–4]. While acute and appropriately resolved inflammation is protective, persistent or dysregulated inflammatory responses drive chronic inflammation, a key contributor to debilitating conditions such as inflammatory bowel disease (IBD), cardiovascular disease, metabolic disorders, neurodegeneration, and cancer, which collectively represent a major burden on global morbidity and mortality [1,2,4,5]. Central signaling pathways, including NF-κB, MAPKs, and JAK/STAT, regulate the expression of potent proinflammatory mediators (e.g., TNF-α, IL-6, iNOS, COX-2) and shape the amplitude and duration of innate immune activation [1,4,6,7].

Recognition of pathogen-associated molecular patterns (PAMPs) by pattern recognition receptors (PRRs) is a major trigger of innate immune responses [1]. For instance, lipopolysaccharide (LPS), a potent endotoxin from Gram-negative bacteria, potently engages Toll-like receptor 4 (TLR4) on macrophages and other innate cells. This interaction leads to significant activation of NF-κB–dependent transcription, driving the expression of proinflammatory cytokines and effector enzymes [1,2,8]. Given the central role of macrophage-derived mediators in initiating and driving tissue pathology, the LPS-stimulated RAW 264.7 macrophage model remains a highly relevant experimental system for evaluating anti-inflammatory interventions. This model allows for the assessment of crucial inflammatory markers such as nitric oxide (NO) production and the expression of iNOS, COX-2, and various cytokines [1,3,4].

Conventional anti-inflammatory drugs, including non-steroidal anti-inflammatory drugs (NSAIDs) and corticosteroids, provide symptomatic relief in many inflammatory states. However, their use is often constrained by significant limitations, including adverse effects like gastrointestinal irritation, cardiovascular complications, renal dysfunction, and hepatotoxicity, along with suboptimal efficacy in chronic conditions, especially with prolonged use [1,3,4,9]. A pressing need for other options has motivated extensive exploration of alternative therapeutic approaches with distinct mechanisms of action and improved safety profiles, often sourced from natural products [1]. Among these, bioactive peptides (BAPs), also known as anti-inflammatory peptides (AIPs), have emerged as a particularly attractive class of candidates [1,2]. These short amino acid sequences (typically 2–20 amino acids) can be derived from diverse biological sources (e.g., herbal, mammalian, bacterial, marine, food or animal proteins, edible insects, agroindustry by-products) or rationally designed [1,2,4,10,11]. BAPs are characterized by their small size, high specificity, diverse functionalities (e.g., anti-hypertensive, antioxidant, anti-diabetic, anticancer, antimicrobial, and immunomodulatory activities), and generally low toxicity, making them promising alternatives to conventional drugs [1,2,4,10,11]. Their physicochemical properties, including sequence, charge, and hydrophobicity, can be precisely tuned to modulate immune signaling, interact directly with microbial components (such as LPS), and achieve favorable cell selectivity and potency against inflammatory pathways [1,4].

A significant advantage of many BAPs, particularly those with cationic properties and enriched in hydrophobic (Val, Ile, Pro) and positively charged (His, Arg, Lys) amino acids, is their inherent multi-functionality, often exhibiting both potent antimicrobial and anti-inflammatory properties [1,2]. This synergy is highly relevant since bacterial infections and endotoxins like LPS frequently triggers inflammation. For instance, antimicrobial peptides (AMPs) can directly neutralize LPS, preventing its interaction with host TLR4 receptors, and can also modulate immune cell responses by interfering with intracellular inflammatory signaling networks [1,4,8,12]. This dual capacity to combat pathogens and regulate inflammatory response makes them promising therapeutic candidates.

Crocodilians inhabit microbe-rich aquatic environments and frequently sustain severe tissue injuries during territorial combat and predation. However, these wounds heal rapidly and rarely become infected, reflecting an exceptionally robust innate immune defense [13,14]. Hemoglobin-derived fragments isolated from *Crocodylus siamensis* blood have been shown to possess intrinsic antimicrobial and anti-inflammatory properties, together with favorable host-cell cytocompatibility [13,14]. These unique evolutionary adaptations make crocodile hemoglobin an attractive biological template for designing synthetic peptides with combined antimicrobial and anti-inflammatory potential. Building on this rationale, IL15.3 (sequence: IKHWKKVWKHAKKVL) is a 15-residue cationic peptide rationally designed from such a hemoglobin-derived template. The initial template peptide, QL17 (sequence: QAIIHNEKVQAHGKKVL), was truncated to IL15 (sequence: IIHNEKVQAHGKKVL). These peptides are known for their antimicrobial and anti-inflammatory properties [13,14]. Subsequent modifications were applied to IL15, specifically engineered to increase cationicity and enhance hydrophobicity, leading to the development of IL15.3, which demonstrated potent, cell-selective antibacterial activity [15]. Given that many antimicrobial and cationic peptides can interfere with LPS-driven macrophage activation and modulate intracellular inflammatory networks, IL15.3 stands as a strong candidate, exhibiting significant anti-inflammatory activity alongside its known antimicrobial properties.

Using LPS-stimulated RAW 264.7 macrophages, this study measured nitric oxide (NO) production inhibition and analyzed the transcriptional regulation of key pro-inflammatory genes (*iNOS*, *COX-2*, *TNF-α*, and *IL-6*) via real-time PCR. To elucidate underlying molecular mechanisms, targeted gene expression analyses were combined with proteomic profiling to assess the impact of the peptide on inflammatory signaling pathways. This research aims to clarify whether IL15.3 can act as a multifunctional anti-inflammatory lead molecule suitable for further preclinical development in inflammation-related disorders.

## 2. Materials and methods

### 2.1. Peptide synthesis

The IL15.3 peptide (sequence: IKHWKKVWKHAKKVL; net charge +6; hydrophobicity 47%), previously designed and characterized by Sosiangdi et al. [15] was commercially synthesized by GL Biochem Ltd. (Shanghai, China). Synthesis was performed via standard solid-phase peptide synthesis (SPPS) using a fluorenylmethyloxycarbonyl (Fmoc) strategy. The final product was purified to >95% purity by reverse-phase high-performance liquid chromatography (RP-HPLC), and its molecular weight was verified by electrospray ionization mass spectrometry (ESI-MS). For structural visualization, the helical wheel projection and predicted secondary structure illustrating the amphipathic α-helical characteristics of IL15.3 are presented in S1 Fig, generated using the HeliQuest and PEP-FOLD servers, respectively, with graphical rendering performed in PyMOL.

### 2.2. Cell viability assay

Cell viability was determined using the 3-(4,5-dimethylthiazol-2-yl)-2,5-diphenyl tetrazolium bromide (MTT) assay, with modifications based on Phosri et al. [16]. Murine macrophage (RAW 264.7), Normal Human Dermal Fibroblasts (NHDF), and human keratinocyte (HaCaT) cells were cultured in Dulbecco’s Modified Eagle’s Medium (DMEM) supplemented with 10% (v/v) fetal bovine serum and 1% antibiotic-antimycotic (Gibco, USA). Cells were maintained at 37°C in a CO_2_ incubator with 5% CO_2_ and 95% humidity. For the assay, cells were seeded into 96-well plates: RAW 264.7 at 2 × 10^4^ cells/well, HaCaT at 1.5 × 10^4^ cells/well, and NHDF at 8 × 10^3^ cells/well, each in 100 μL of medium. After 24 h of initial incubation at 37°C, the medium was removed and replaced with fresh medium containing IL15.3 at final concentrations ranging from 25 to 200 µg/mL. Following another 24 h incubation at 37°C, the medium was replaced with 100 µL of MTT solution (0.5 mg/mL) and incubated in a CO_2_ incubator at 37°C for 30 min. Subsequently, the medium was carefully removed, and the MTT formazan product was solubilized in 100 µL of DMSO. Absorbance was then measured at 570 nm. Cell viability was calculated using the following equation:


$$\begin{array}{c} \%\text{ Cell viability}=\left(\frac{{\text{A5}\text{7}\text{0}}_{\text{test}}}{{\text{A5}\text{7}\text{0}}_{\text{control}}}\right)\times \text{1}\text{0}\text{0} \end{array}$$

### 2.3. Anti-inflammatory assay

Anti-inflammatory activity was evaluated by measuring nitric oxide (NO) production from RAW 264.7 cells stimulated with lipopolysaccharide (LPS). NO production was quantified using the Griess diazotization reaction, modified from the method of Wanthong et al. [12]. RAW 264.7 cells were seeded at a density of 2 × 10^4^ cells/well in 96-well plates (total volume 100 μL/well) and incubated at 37°C in a humidified atmosphere of 5% for 24 h. Subsequently, cells were stimulated with 100 ng/mL LPS and treated with or without IL15.3 at final concentrations of 50–200 µg/mL, then incubated at 37°C for 24 h. The culture medium was then collected and mixed with Griess reagent at a 1:1 ratio, followed by incubation for 10 min in the dark. Absorbance was measured at 540 nm, while cell viability was assessed using the MTT assay. NO production was calculated using the following equation:


$$\begin{array}{c} \%\text{ NO production}=\left(\frac{{\text{A540}}_{\text{test}}}{{\text{A540}}_{\text{control}}}\right)\times \text{1}\text{0}\text{0} \end{array}$$

### 2.4. Measurement of intracellular reactive oxygen species (ROS)

The production of intracellular ROS was evaluated using the 2′,7′-dichlorodihydrofluorescein diacetate (DCFH-DA) fluorescence indicator. RAW 264.7 cells were seeded at a density of 2 × 10^4^ cells/well in 96-well plates (total volume 100 μL/well) and incubated at 37°C in a humidified atmosphere of 5% for 24 h. After the initial incubation, cells were stimulated with 100 ng/mL LPS and treated with or without IL15.3 at final concentrations of 50–200 µg/mL. Following a 24 h incubation period, the cells were stained with 50 µM DCFH-DA in fetal bovine serum (FBS) free medium (50 µL/well) in the dark. The plates were further incubated for 30 min at 37°C. Subsequently, the staining solution was removed, and the cells were washed three times with 100 µL of 1 × phosphate-buffered saline (PBS) to remove extracellular dye. Fluorescence images were captured at 20 × magnification using an inverted microscope (EVOS™ M5000, Thermo Scientific, USA). The relative fluorescence intensity, representing intracellular ROS levels, was quantified using ImageJ software (version 1.54g, USA).

### 2.5. Proteomic analysis

#### 2.5.1. Protein sample preparation.

RAW 264.7 cells were seeded at a density of 2 × 10^5^ cells/well in 12-well plates (1,000 μL/well) and treated as described in the Anti-inflammatory assay section. (LPS ± IL15.3). Following treatment, cells were harvested, and total proteins were extracted using a 0.5% sodium dodecyl sulfate (SDS) solution, a method modified from Maijaroen et al. [10]. Cell lysates were centrifuged at 10,000 rpm for 10 min at 4°C to remove cellular debris, and the resulting supernatant was collected. Protein in the supernatant were then precipitated by adding two volumes of cold acetone containing 0.1 mM dithiothreitol (DTT), followed by overnight incubation at −20°C. Precipitated proteins were pelleted by centrifugation at 10,000 rpm for 10 min, and after the acetone was evaporated off, the pellet was re-dissolved in 0.5% SDS. Protein concentration was then measured using the Lowry method.

For trypsin digestion, 5 µg of each protein sample was first reduced with 10 mM DTT in 10 mM ammonium bicarbonate to break sulfhydryl bonds, then alkylated with 30 mM iodoacetamide (IAA) in 10 mM ammonium bicarbonate to prevent disulfide bond reformation. The alkylation reaction was performed at room temperature for 60 min in the dark. Proteins were then digested overnight at 37°C using sequencing-grade trypsin (Promega, Germany), prepared as a 10 ng/µL solution in 50% acetonitrile/10 mM ammonium bicarbonate, at a protein-to-trypsin ratio of 20:1 (w/w). The resulting tryptic peptides were dried under vacuum at 44°C and subsequently reconstituted in 0.1% formic acid prior to injection into the liquid chromatography–tandem mass spectrometry (LC-MS/MS) system for analysis.

#### 2.5.2. Label-free LC-MS/MS.

Tryptic peptides were analyzed on an Ultimate3000 Nano/Capillary LC System (Thermo Scientific, UK) coupled to a ZenoTOF 7600 mass spectrometer (SCIEX, Framingham, MA, USA). Briefly, 100 ng of each peptide digest was first enriched on a µ-Precolumn (300 µm i.d. × 5 mm; Pepmap 100 resin, 5 µm, 100 Å; Thermo Scientific, UK). Peptides were then separated on an IonOpticks Aurora Elite XS C18 column (150 mm × 75 µm i.d., 1.7 µm, 120 Å) housed in a column oven maintained at 60°C. Mobile phases consisted of solvent A (0.1% formic acid in water) and solvent B (0.1% formic acid in 80% acetonitrile), delivered at a constant flow rate of 0.30 µL/min using a 5–55% solvent B gradient over 30 min.

The ZenoTOF 7600 source and gas parameters were set as follows: ion source gas 1, 8 psi; curtain gas, 35 psi; CAD gas, 7 psi; source temperature, 200°C; positive polarity; and spray voltage, 3300 V. Data-dependent acquisition (DDA) was performed by selecting the top 50 most abundant precursor ions from each survey MS1 scan for MS/MS, using an intensity threshold above 150 cps. Precursor ions were dynamically excluded for 12 s after two MS/MS events, with dynamic collision energy enabled. MS2 spectra were acquired over 100–1,800 m/z with a 50 ms accumulation time and the Zeno trap enabled, using a declustering potential of 80 V (no DP spread) and a collision energy spread of 0 V. Time bins were summed across all channels using a 150,000 cps Zeno trap threshold, giving a total cycle time of 3.0 s for the Top 60 DDA method.

#### 2.5.3. Proteomic data processing and analysis.

Protein quantitation for individual samples was performed using MaxQuant (version 2.5.0.0) with the integrated Andromeda search engine [17]. MS/MS spectra were searched against the UniProt *Mus musculus* database, and label-free quantification was carried out using MaxQuant’s standard settings. Key search parameters included trypsin as the digesting enzyme, up to two missed cleavages, and a mass tolerance of 20 ppm for the main search. Carbamidomethylation of cysteine was specified as a fixed modification, while oxidation of methionine and acetylation of the protein N-terminus were included as variable modifications, with a maximum of five modifications allowed per peptide. Potential contaminants were automatically included in the search space via the contaminants.fasta file provided by MaxQuant. For protein identification, only peptides of at least 7 amino acids were considered, and protein groups were required to have at least one unique peptide. Proteins carried forward for further analysis required a minimum of two peptides in total, including at least one unique peptide. The protein false discovery rate (FDR) was set at 1%, estimated using a reversed decoy database. Following identification, protein relationships were further elucidated through the construction of a Venn diagram, and finally, identified proteins were functionally classified and categorized into specific pathways using the publicly available Protein ANalysis THrough Evolutionary Relationships (PANTHER) database.

### 2.6. Gene expression analysis using real-time PCR

RAW 264.7 cells were stimulated with LPS and treated with or without IL15.3 (final concentrations ranging from 50 to 200 µg/mL) for 24 h. After incubation, the culture medium was collected for NO production measurement using the Griess reagent, as described in the previous Anti-inflammatory assay section. The remaining cells were then harvested and processed for total RNA extraction using Trizol reagent (Invitrogen, USA) according to the manufacturer’s protocol. Complementary DNA (cDNA) was synthesized from the extracted RNA using the RevertAid First Strand cDNA Synthesis Kit (Thermo Fisher Scientific Inc, USA). Quantitative real-time PCR (qPCR) was performed using a LightCycler 480 instrument (Roche, Switzerland). Each PCR reaction mixture (20 μL total volume) contained 2 μL of cDNA, 10 μL of LightCycler® SYBR® Green I Master mix (Roche, Switzerland), and 1 μL of each gene-specific primer (10 μM stock concentration). Annealing temperatures for each primer pair were determined individually and are listed in Table 1. Gene expression levels were quantified using the comparative 2^−ΔΔCT^ method, with *β-actin* serving as the internal reference gene for normalization.

**Table 1:** Primer sequences for real-time PCR.

Gene	Primer sequences (5′ to 3′)		Product size (bp)	References
*iNOS*	Forward	5′-TTTCCAGAAGCAGAATGTGACC-3′	294	NM_010927.4
	Reverse	5′-AACACCACTTTCACCAAGACTC-3′		
*COX-2*	Forward	5′-GAAATATCAGGTCATTGGTGGAG-3′	237	NM_011198.5
	Reverse	5′-GTTTGGAATAGTTGCTCATCAC-3′		
*TNF-α*	Forward	5′-CCACGCTCTTCTGTCTACTG-3′	169	NM_013693.3
	Reverse	5′-ACTTGGTGGTTTGCTACGAC-3′		
*IL-6*	Forward	5′-CTGATGCTGGTGACAACCAC-3′	143	NM_031168.2
	Reverse	5′-TCCACGATTTCCCAGAGAAC-3′		
*STAT1*	Forward	5′-GAAGAATCCACCAACGG-3′	200	NM_001357627.1
	Reverse	5′-GAGATCACCACGACAGG-3′		
*STAT3*	Forward	5′-ACCTCCAGGACGACTTTGAT-3′	204	NM_213659.4
	Reverse	5′-GTGTCTTCTGCACGTACTCCA-3′		
*NF-κB*	Forward	5′-GGCCTGCAAAGGTTATCGTT-3′	300	NM_001410442.1
	Reverse	5′-TGTCTGTGAGTTGCCGGTCTT-3′		
*JNK*	Forward	5′-GCCACAAAATCCTCTTTCCA-3′	176	NM_001310454.2
	Reverse	5′-CACATCGGGGAACAGTTTCT-3′		
*Keap1*	Forward	5′-AGCGTGGAGAGATATGAGCC-3′	185	NM_001110307.1
	Reverse	5′-ATCATCCGCCACTCATTCCT-3′		
*Nrf2*	Forward	5′-ACATGGAGCAAGTTTGGCAG-3′	234	NM_010902.4
	Reverse	5′-TGGAGAGGATGCTGCTGAAA-3′		
*β-actin*	Forward	5′-GCTACAGCTTCACCACCACA-3′	208	NM_007393.5
	Reverse	5′-AAGGAAGGCTGGAAAAGAGC-3′		

### 2.7. Molecular docking analysis

The IL15.3 peptides were docked onto iNOS and the Kelch domain of Kelch-like ECH-associated protein 1 (Keap1) using the web-based HPEPDOCK server [18], with peptide sequences entered as FASTA-format ligands. The receptor structures used were the crystal structures of iNOS (PDB ID: 1M9T) [19] and Keap1 (PDB ID: 7Q6S) [20]. For each resulting peptide–protein complex, binding free energies was calculated via the HawkDock server [21]. The peptide–protein interaction profiles, including identification of hydrogen bonds, hydrophobic contacts, salt bridges, and π-cation interactions, was analyzed with the Protein–Ligand Interaction Profiler (PLIP; https://plip-tool.biotec.tu-dresden.de/plip-web/plip/index). Finally, PyMOL (Schrödinger LLC) was used to generate three-dimensional visualizations of how the peptides were oriented within iNOS and Keap1 binding pocket.

### 2.8. Statistical analysis

Statistical analysis was performed using One-way ANOVA and Duncan’s new multiple range test with SPSS version 19.0 (IBM SPSS Statistics Software, International Business Machines (IBM) Corporation, New York, USA). A *p*-value of < 0.05 indicated a statistically significant difference.

## 3. Results and discussion

### 3.1. Cell viability

Cell viability, indicating the cytotoxic effects of IL15.3, was assessed in RAW 264.7 macrophages, normal human dermal fibroblasts (NHDF), and human keratinocytes (HaCaT) using the MTT assay. This colorimetric assay measures cellular metabolic activity through the reduction of yellow tetrazolium salt to purple formazan crystals by mitochondrial succinate dehydrogenase and other reductases in viable cells [22,23]. The formazan product is quantified spectrophotometrically, with absorbance directly proportional to cell viability [22,23]. At concentrations ranging from 25 to 200 µg/mL, IL15.3 exhibited low toxicity towards both RAW 264.7 and NHDF cells, with cell viability consistently maintained above 90% (Fig 1A and 1B). For HaCaT cells, IL15.3 also demonstrated low toxicity, maintaining viability above 90% at concentrations between 25 and 100 µg/mL. However, at the highest concentration tested (200 µg/mL), the viability of HaCaT cells decreased slightly to 84.60% (Fig 1C). The concentration range of 25–200 µg/mL was selected based on our previous characterization, which demonstrated that IL15.3 possessed potent antimicrobial efficacy (MIC = 4–12 µg/mL), low cytotoxicity (IC_50_ > 200 µg/mL, HC_50_ > 2,000 µg/mL), and the highest selectivity index among designed variants [15]. Consistently, current MTT assays confirmed >80–90% cell viability across RAW 264.7, NHDF, and HaCaT cells within this range, establishing it as a safe window for anti-inflammatory evaluation. This favorable profile aligns with the design principle of selective antimicrobial peptides, which are engineered to exploit differences between microbial and mammalian membranes to ensure high selective toxicity toward pathogens while maintaining low cytotoxicity in host cells [15]. Nevertheless, the cytocompatibility of IL15.3 exhibited subtle cell type-dependent variations, with RAW 264.7 macrophages showing greater tolerance than NHDF and HaCaT cells at higher concentrations. This differential susceptibility likely reflects cell type-specific variations in membrane lipid composition, surface charge, and membrane fluidity.

**Fig 1:**
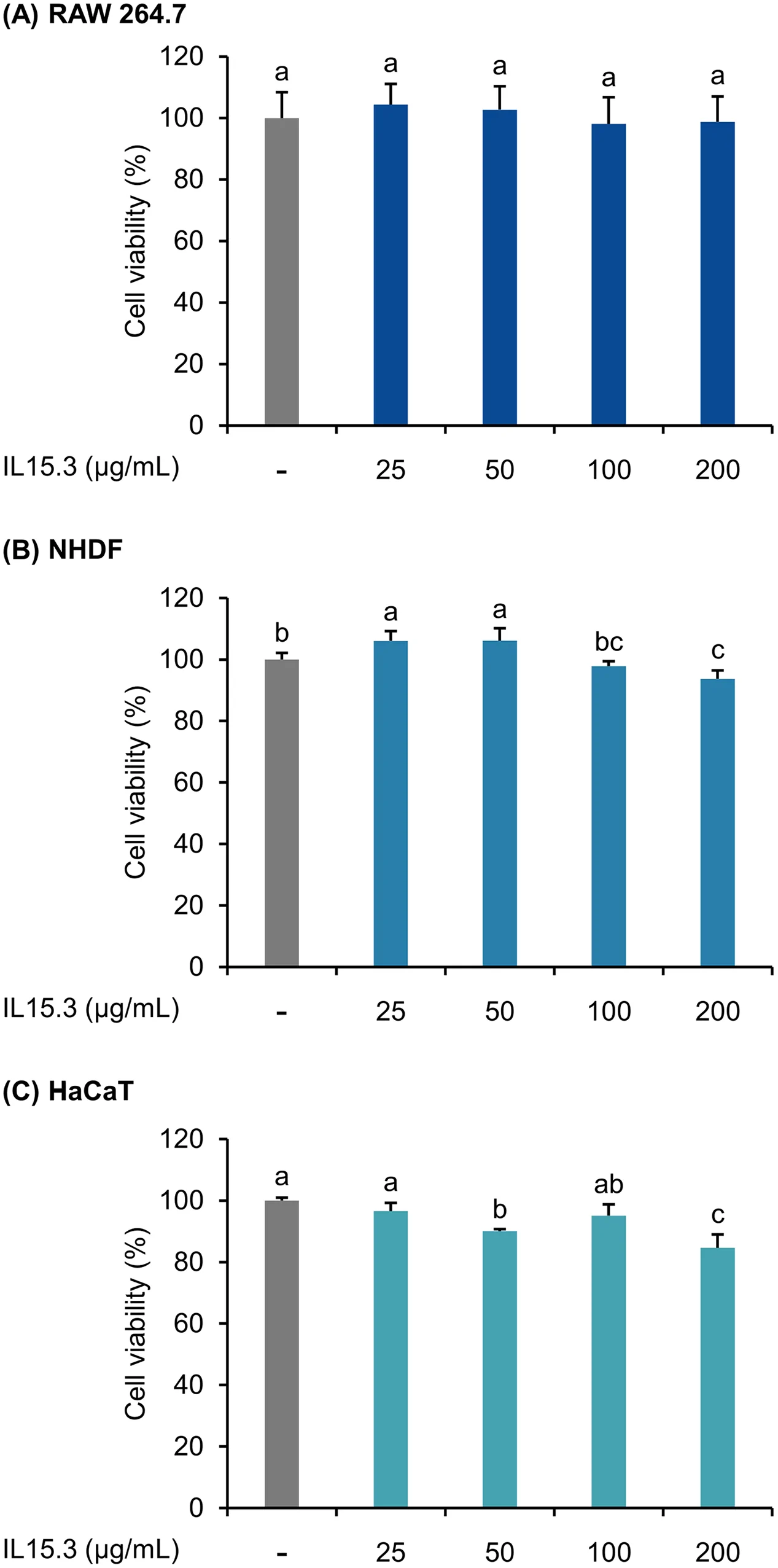
Cytotoxicity profile of IL15.3 peptide. Cell viability of (A) RAW 264.7 macrophages, (B) NHDF cells, and (C) HaCaT keratinocytes following treatment with IL15.3 peptide, assessed by MTT assays. Data are presented as mean ± standard deviation. Statistical analysis was performed using one-way ANOVA followed by Duncan’s multiple range test, where different letters indicate significant differences (*p* < 0.05).

### 3.2. The anti-inflammatory effects of IL15.3 on RAW 264.7 cells

The anti-inflammatory effects of IL15.3 were investigated by measuring nitric oxide (NO) production in LPS-stimulated RAW 264.7 macrophages. LPS, a major component of the outer membrane of Gram-negative bacteria, is a potent immunostimulatory molecule that triggers robust inflammatory responses in macrophages [24]. Upon recognition by Toll-like receptor 4 (TLR4), LPS initiates signaling cascades that activate the nuclear factor-kappa B (NF-κB) pathway, leading to upregulation of pro-inflammatory mediators, including inducible nitric oxide synthase (iNOS), which catalyzes NO production [25,26]. In this study, cells stimulated with LPS alone served as the positive inflammatory control, establishing the condition for maximal NO induction (set as 100% NO production). Treatment with IL15.3 significantly and dose-dependently reduced NO production. Specifically, IL15.3 at concentrations of 50, 100, and 200 µg/mL resulted in NO production levels of 86.79%, 76.70%, and 58.00%, respectively, relative to the LPS-stimulated control (Fig 2A). This indicates a clear anti-inflammatory effect as the peptide concentration increased.

**Fig 2:**
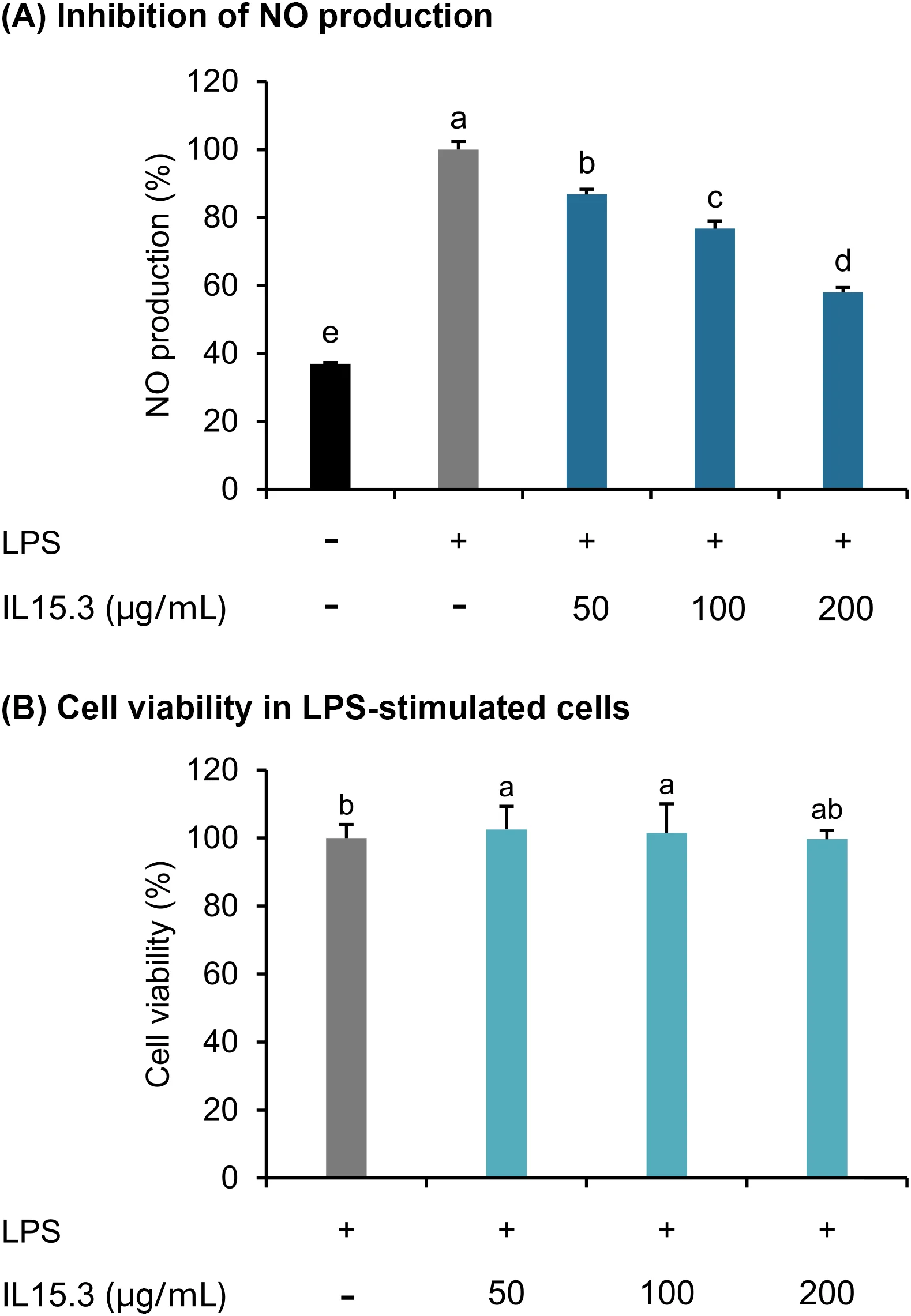
Anti-inflammatory activity and safety profile of IL15.3 in LPS-stimulated RAW 264.7 macrophages. (A) Dose-dependent reduction of nitric oxide (NO) production (expressed as % of LPS control) and (B) corresponding cell viability were measured following 24 h of treatment with 100 ng/mL LPS in the presence or absence of IL15.3 (50–200 µg/mL). Data are presented as mean ± standard deviation. Statistical analysis was performed using one-way ANOVA followed by Duncan’s multiple range test, where different letters indicate significant differences (*p* < 0.05).

To confirm that the observed reduction in NO production was due to anti-inflammatory activity rather than cytotoxicity, cell viability was simultaneously assessed in the LPS-stimulated RAW 264.7 cells treated with IL15.3. At all tested concentrations (50–200 µg/mL), IL15.3 maintained high cell viability (>90%), demonstrating no significant cytotoxicity that would falsely contribute to the decrease in NO levels (Fig 2B). These findings collectively confirm that IL15.3 exerts anti-inflammatory effects by attenuating NO release in activated macrophages.

The anti-inflammatory activity of IL15.3 (IKHWKKVWKHAKKVL) is supported by its physicochemical features, which resemble motifs found in known bioactive anti-inflammatory peptides [27,28]. First, IL15.3 contains six lysine and two histidine residues, resulting in a net charge of +6 at physiological pH. This strong cationicity promotes electrostatic interactions with anionic inflammatory mediators and cell membranes [27]. Importantly, the choice of lysine over arginine as the principal basic residue was guided by the lysine-rich *C. siamensis* hemoglobin template [13–15] and by a strategy to minimize host toxicity. Unlike the resonance-stabilized guanidinium group of arginine, which can promote multivalent membrane interactions and cytotoxicity, the primary amine of lysine provides comparable electrostatic interactions while better preserving mammalian cell safety [15,29]. In addition, IL15.3 also displays an amphipathic structure featuring a hydrophobic N-terminal motif (Ile-Lys-His-Trp). The presence of isoleucine at the N-terminus is particularly notable, as similar sequences (such as Ile-Ala, Ile-Pro-Pro, Ile-Arg-Trp, and Ile-Gln-Trp) are associated with potent anti-inflammatory properties [27,28]. Together, these structural features likely contribute to the observed anti-inflammatory effects of IL15.3.

### 3.3. Synthetic peptides IL15.3 attenuate LPS-induced ROS production

LPS serves as a primary stimulus for inflammatory activation in macrophages and is also a potent trigger for the generation of reactive oxygen species (ROS) [30,31]. The accumulation of intracellular ROS is a pivotal event in the inflammatory activation of macrophages, as these two processes are intricately linked through shared signaling cascades [30,31]. In this study, RAW 264.7 macrophages stimulated with 100 ng/mL LPS exhibited a dramatic increase in green fluorescence compared to untreated control cells, indicating a substantial accumulation of intracellular ROS (Fig 3A). These observations align with established paradigms where LPS initiates cascades such as the NF-κB and MAPK pathways, which collectively drive the upregulation of pro-inflammatory mediators and oxidative stress markers [8,32]. Notably, co-treatment with the synthetic peptide IL15.3 at 50, 100, and 200 µg/mL resulted in a concentration-dependent reduction in DCF fluorescence intensity. Specifically, the average ROS intensity decreased from approximately 18.02 arbitrary units in the LPS-only group to 4.67 arbitrary units in the group co-treated with 200 µg/mL IL15.3 (Fig 3A and 3B). Visual evidence from the micrographs, supported by quantitative analysis, suggests that IL15.3 effectively attenuates LPS-induced ROS accumulation. This antioxidant effect likely correlates with our transcriptional data, which showed that IL15.3 significantly suppresses the expression of key pro-inflammatory genes, including *iNOS*, *COX-2*, *TNF-α*, and *IL-6*.

**Fig 3:**
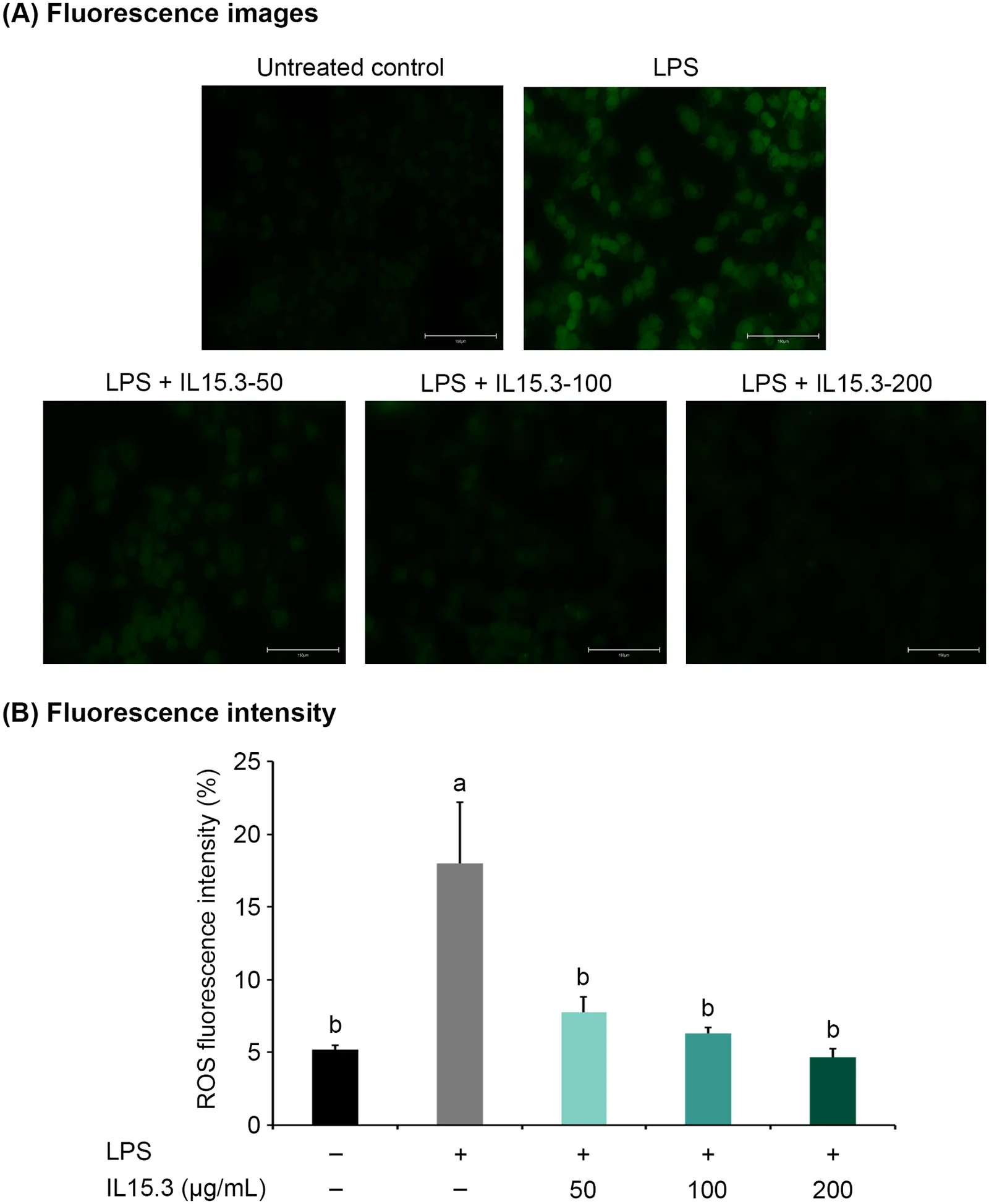
IL15.3 peptide suppresses intracellular ROS accumulation in LPS-stimulated RAW 264.7 macrophages. (A) Representative fluorescence microscopy images of RAW 264.7 cells stained with DCFH-DA after 24 h of treatment. Cells were either untreated or exposed to 100 ng/mL LPS alone or in combination with IL15.3 at 50 (IL15.3-50), 100 (IL15.3-100), and 200 (IL15.3-200) µg/mL. Intracellular ROS accumulation is represented by green fluorescent signals. All images were captured at 20 × magnification (scale bar = 150 μm). (B) Mean fluorescence intensity of intracellular ROS was quantified using ImageJ software. Data are presented as mean ± standard deviation from three independent experiments. Statistical analysis was performed using one-way ANOVA followed by Duncan’s multiple range test, where different letters indicate significant differences (*p* < 0.05).

### 3.4. Label-free proteomics elucidates the multi-target anti-inflammatory mechanism of IL15.3

#### 3.4.1. Proteomic profiling of LPS-stimulated RAW 264.7 cells treated with IL15.3.

To gain a comprehensive understanding of the molecular mechanisms underlying the anti-inflammatory effects of IL15.3, label-free quantitative proteomic analysis was performed on RAW 264.7 macrophages under various conditions. A total of 5,191 proteins were classified and profiled using liquid chromatography-mass spectrometry (LC-MS). The experimental groups included: untreated cells, LPS-stimulated cells (LPS), and LPS-stimulated cells co-treated with IL15.3 at 50 µg/mL (IL15.3–50), 100 µg/mL (IL15.3–100), and 200 µg/mL (IL15.3–200). The total protein counts identified in each group were 2,959 (Untreated), 2,992 (LPS), 2,214 (IL15.3–50), 2,728 (IL15.3–100), and 2,859 (IL15.3–200), respectively (Fig 4). The overlap and unique protein profiles across these conditions were visualized using a Venn diagram generated by InteractiVenn.

**Fig 4:**
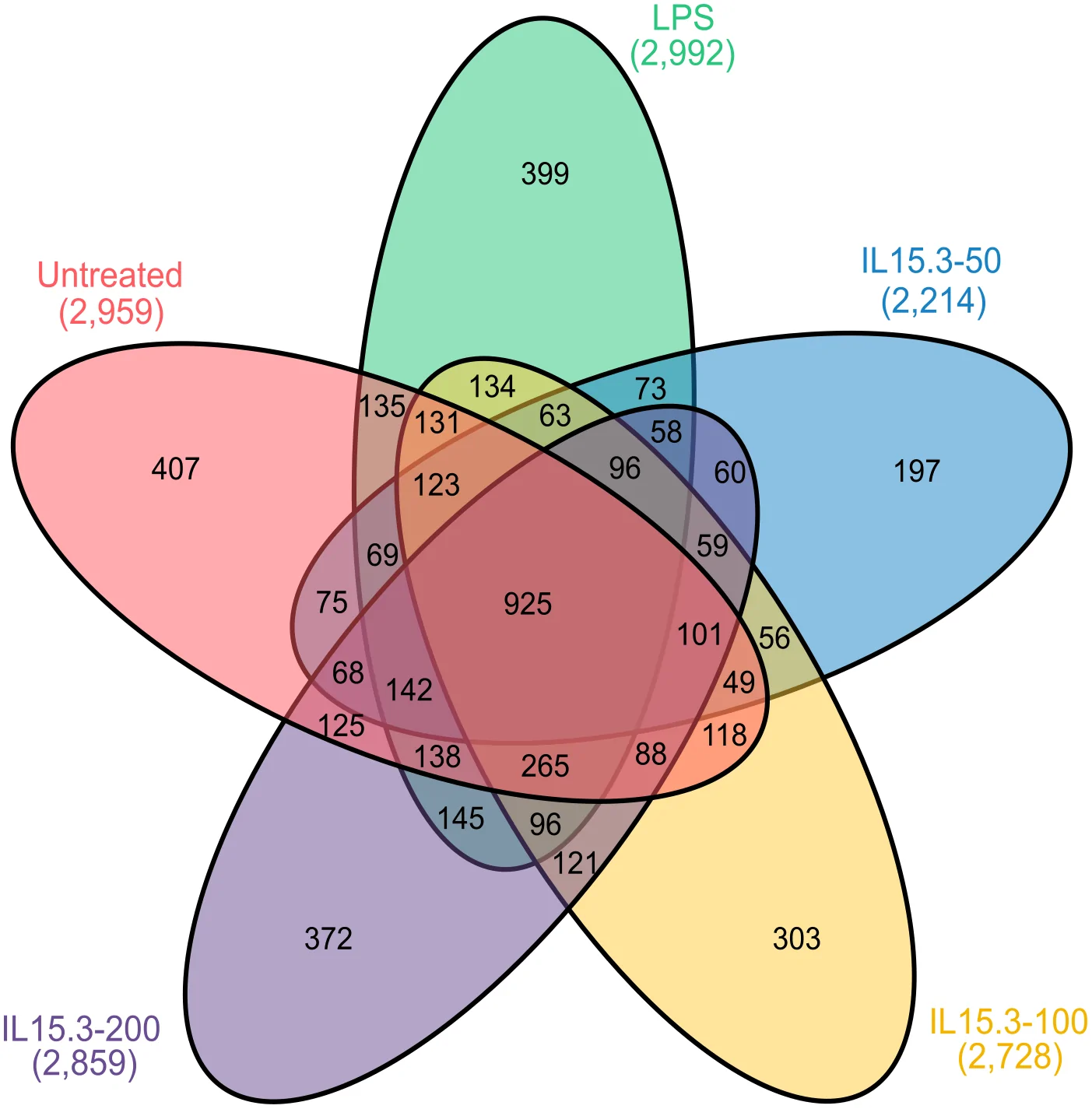
Venn diagram illustrating the overlap and uniqueness of proteins identified in RAW 264.7 macrophages subjected to different treatments, generated using InteractiVenn. Groups include untreated control (untreated: 2,959 proteins), LPS-stimulated cells (LPS: 2,992 proteins), and LPS-stimulated cells co-treated with IL15.3 at 50 µg/mL (IL15.3-50: 2,214 proteins), 100 µg/mL (IL15.3-100: 2,728 proteins), and 200 µg/mL (IL15.3-200: 2,859 proteins).

Functional annotation of the 59 proteins consistently identified across all IL15.3-treated groups (IL15.3–50, IL15.3–100, and IL15.3–200) was performed using the Panther Classification System (v. 19.0) and the *Mus musculus* gene database to reveal their molecular roles (Fig 5). This comprehensive analysis characterized these proteins based on molecular function, biological process, cellular component, pathway, and protein class. Analysis of molecular function (Fig 5A) demonstrated the dominance of binding (40.0%), catalytic activity (26.0%), and molecular function regulator activity (10.0%), indicating the influence of IL15.3 on diverse cellular interactions and regulatory processes. For biological processes (Fig 5B), proteins were largely enriched in cellular process (31.2%), biological regulation (18.3%), and metabolic process (10.8%), with a notable 4.3% involvement in immune system process and biological process involved in interspecies interactions between organisms, highlighting relevance to host defense. Cellular component analysis (Fig 5C) showed widespread localization, mainly within cellular anatomical entity (82.6%), suggesting broad subcellular effects. Crucially, pathway analysis (Fig 5D) revealed significant enrichment in the Inflammation mediated by chemokine and cytokine signaling pathway (18.8%), followed by Gonadotropin-releasing hormone receptor pathway (12.5%), Ionotropic glutamate receptor pathway (12.5%), and Huntington disease (12.5%). This directly implicates IL15.3 in inflammatory cascades, among other biological pathways. Lastly, common protein classes (Fig 5E) included gene-specific transcriptional regulator (16.7%), protein modifying enzyme (14.6%), and metabolite interconversion enzyme (12.5%). These comprehensive functional classifications underscore that IL15.3 modulates a wide array of molecular targets and biological processes, with particular engagement in immune system processes and key inflammatory signaling pathways, further supported by the identification of specific anti-inflammatory proteins detailed in Table 2.

**Table 2:** Anti-inflammatory mediators exclusively expressed in LPS-stimulated RAW 264.7 cells following treatment with IL15.3.

Entry Name	Gene Name	Protein Name	Protein Function	References
ISG15	Isg15	Ubiquitin-like protein ISG15	Promotes anti-inflammatory cytokines (e.g., IL-10, TGF-β) and suppresses pro-inflammatory mediators via MAPK/PI3K-dependent pathways.	[33,34]
Q9QX52	Pex14	Peroxisomal membrane protein PEX14 (Peroxin-14)	Supports peroxisome biogenesis, fatty acid oxidation, ROS detoxification, and anti-inflammatory responses.	[35,36]
Q3MI11	Tnip3 (ABIN-3)	Tnip3 protein	Inhibits NF-κB and PI3K/Akt pathways, suppressing pro-inflammatory cytokines (e.g., IL-1β, IL-6, TNF-α).	[37,38]
PRGR	Pgr	Progesterone receptor (PR)	Transrepresses NF-κB/TLR signaling; suppresses iNOS, COX-2, and pro-inflammatory cytokines; promotes IL-10 and TGF-β.	[39]

**Fig 5:**
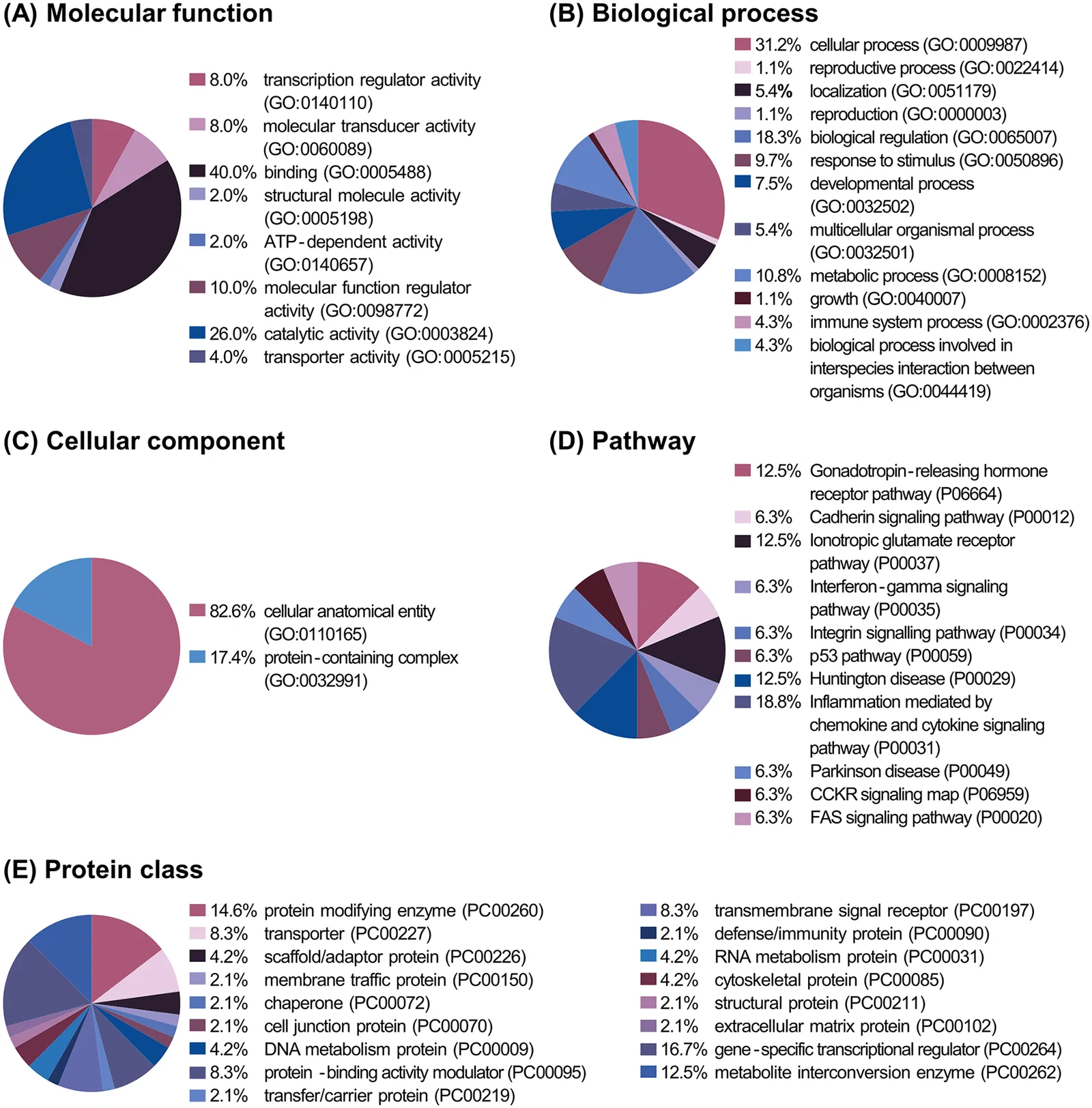
Functional classification of 59 proteins commonly identified in LPS‑stimulated RAW 264.7 cells co‑treated with IL15.3 (50, 100, and 200 µg/mL). Annotations were assigned using the PANTHER Classification System (version 19.0) and grouped as: (A) molecular function, (B) biological process, (C) cellular component, (D) pathways, and (E) protein class. Percentages indicate the proportion of the 59 proteins assigned to each category.

#### 3.4.2. Upregulation of anti-inflammatory proteins exclusively expressed by IL15.3 treatment.

Among the 59 proteins commonly expressed across all IL15.3-treated groups, four were identified as having documented anti-inflammatory functions (Table 2). These included Ubiquitin-like protein ISG15 (ISG15), Peroxisomal membrane protein PEX14 (PEX14), TNFAIP3-interacting protein 3 (TNIP3/ABIN-3), and Progesterone receptor (PR). Importantly, these proteins were not detected, or were below the proteomic detection threshold, in LPS-stimulated and untreated control samples. The continual detection of these proteins across the tested IL15.3 concentrations is consistent with induction by IL15.3 and with a potential role for these factors in the peptide-mediated attenuation of LPS-induced inflammatory responses. Each has mechanistic plausibility for contributing to the anti-inflammatory phenotype observed in other assays.

ISG15 is a strong mechanistic candidate because it has well-documented anti-inflammatory and immunomodulatory effects [33,34]. It suppresses inflammation by inhibiting NF-κB activation, leading to reduced production of pro-inflammatory cytokines such as IL-1β and IL-6. Studies show that loss of ISG15 increases NF-κB activity and cytokine levels in macrophages, while increasing ISG15 leads to their suppression [34]. ISG15 also boosts the expression of anti-inflammatory cytokines, including IL-10, TGF-β, IL-35, IL-37, and IL-38 [33,34]. The consistent enrichment of ISG15 in IL15.3-treated samples therefore suggests the peptide may act, at least in part, by upregulating ISG15 in macrophages, linking NF-κB suppression to enhanced anti-inflammatory signaling and helping to explain its broad effects in LPS-challenged cells.

The exclusive detection of PEX14 in IL15.3-treated macrophages (Table 2) suggests that IL15.3 may trigger anti-inflammatory effects mediated by peroxisomes. PEX14 is essential for peroxisome function, enabling fatty acid β-oxidation and redox balance, which helps neutralize reactive oxygen species (ROS). Consistent with an anti-inflammatory role, Pex14 knockdown strongly increased LPS-induced COX-2 and TNF-α, whereas induction of peroxisome biogenesis blocked COX-2 upregulation in both mouse and human macrophages [35,36].

TNIP3, also known as ABIN-3, functions as a negative regulator of inflammatory signaling. TNIP3 inhibits both the NF-κB and PI3K/Akt signaling pathways, thereby suppressing the expression of pro-inflammatory cytokines, including IL-1β, IL-6, and TNF-α in LPS-activated cells [37,38]. The exclusive detection of TNIP3 in IL15.3-treated cells suggests that IL15.3 may suppress NF-κB-dependent genes (*iNOS*, *COX-2, TNF-α*, *IL-6*) observed in our gene expression analysis.

PR is a nuclear hormone receptor that, when activated by progesterone or related ligands, can move into the nucleus and directly interfere with inflammatory signaling pathways, particularly NF-κB and Toll-like receptor (TLR) signaling [39]. In macrophages, PR activation suppresses the expression of key pro-inflammatory genes, including *iNOS*, *COX-2*, *TNF-α*, *IL-6*, and *IL-1β*, and promotes the production of anti-inflammatory and tissue-repair mediators such as *IL-10* and *TGF-β* [39].

The exclusive detection of these four anti-inflammatory proteins in IL15.3-treated cells suggests that IL15.3 may promote an anti-inflammatory phenotype in macrophages. Since these proteins are recognized for their roles in suppressing NF-κB, TLR, and cytokine signaling, their presence may contribute to the downregulation of pro-inflammatory genes.

#### 3.4.3. IL15.3 modulates inflammatory regulators shared between LPS and treatment groups.

Of the 96 proteins detected across the LPS control and IL15.3-treated groups (50, 100, and 200 µg/mL), representative examples of inflammation-related proteins are shown in Table 3. Two proteins with anti-inflammatory functions, Pro-neuregulin-1 (NRG-1) and Dab2ip protein (DAB2IP) exhibited elevated expression levels in all IL15.3-treated groups relative to the LPS control.

**Table 3:** Functional overview of select proteins commonly identified across all LPS-treated and IL15.3-co-treated RAW264.7 macrophage groups. Protein abundance is shown as log2 expression.

Entry name	Gene name	Protein name	Expression level (log2)			Protein function	References
			LPS	IL15.3 concentration (µg/mL)			
				50	100			200
A0A5F8MPA3	Nrg1	Pro-neuregulin-1	11.91	12.03	12.28	12.06	Inhibits IκBα phosphorylation/degradation and NF-κB p65 nuclear translocation, reducing LPS-induced TNF-α and IL-6	[40]
Q52KF5	Dab2ip	Dab2ip protein	7.74	8.17	8.68	10.46	Bifunctional tumor suppressor; coordinately suppresses oncogenic and inflammatory signaling pathways.	[41,42]

NRG-1 showed log_2_ expression increases from 11.91 in the LPS group to 12.03, 12.28, and 12.06 with increasing IL15.3 concentrations (Table 3). NRG-1, a neuregulin family growth factor signaling through ErbB receptors, exhibits anti-inflammatory activity in macrophages and other immune cells. NRG-1 inhibits the phosphorylation and degradation of IκB-α, thereby preventing nuclear translocation of NF-κB p65 and reducing transcription of pro-inflammatory genes, including *TNF-α*, and *IL-6* [40]. Similarly, DAB2IP demonstrated substantial upregulation from 7.74 (LPS control) to 8.17, 8.68, and 10.46 across the treatment groups (Table 3). DAB2IP functions as a bifunctional tumor suppressor through two distinct molecular domains that coordinate suppression of oncogenic and inflammatory signaling. Loss of DAB2IP results in simultaneous amplification of multiple pathways critical to cancer development and inflammatory responses [41,42].

Collectively, the robust upregulation of both NRG-1 and DAB2IP suggests that IL15.3 may contribute to the resolution of inflammation by not only impacting immediate inflammatory cascades but also by promoting internal regulatory mechanisms geared toward managing sustained inflammatory pathology

#### 3.4.4. Comprehensive modulation of core inflammatory pathways revealed by analysis of constitutively expressed proteins.

Analysis of the 925 proteins commonly expressed across all five experimental conditions (untreated control, LPS control, and LPS + IL15.3 at 50, 100 and 200 µg/mL) identified seven proteins involved in inflammatory regulation (Table 4). These proteins were categorized into two functional groups: anti-inflammatory regulators and pro-inflammatory mediators.

**Table 4:** Differential expression of constitutively expressed proteins involved in inflammatory signaling pathways in RAW 264.7 macrophages. Protein abundance is presented as log2 expression.

Entry Name	Gene Name	Protein Name	Expression Level (log2)					Protein Function	References
			Untreated	LPS	IL15.3 concentration (µg/mL)				
					50	100	200		
I18BP	Il18bp	Interleukin-18-binding protein (IL-18BP)	6.81	7.58	7.47	8.45	9.65	Binds and neutralizes IL-18, blocking IL-18 receptor signaling and downstream IFN-γ and NF-κB activation.	[43,44]
Q3UXZ1	Socs5	Suppressor of cytokine signaling 5 (SOCS5)	7.53	7.05	6.01	8.78	8.45	Key negative regulator of cytokine signaling, inhibiting the JAK/STAT pathway and restraining uncontrolled immune activation.	[45,46]
IKKB	Ikkb	Inhibitor of nuclear factor kappa-B kinase subunit beta (IKKβ)	10.44	12.91	10.62	10.15	7.85	Catalytic IKK subunit that phosphorylates IκBα to activate NF-κB–dependent inflammatory gene expression.	[47]
Q3URU8	Jak1	Tyrosine-protein kinase JAK1	10.44	11.52	9.44	7.66	9.96	Transduces signals from pro-inflammatory cytokines by phosphorylating STAT proteins, driving transcription of inflammatory genes.	[6,48]
P97423	Jak3	Tyrosine-protein kinase JAK3	12.36	10.63	8.88	7.81	7.31	Mediating γc cytokine receptor signaling; important for lymphocyte activation and inflammatory responses.	[6,48,49]
Q3TQZ7	Mapk10	Stress-activated protein kinase JNK (EC 2.7.11.24)	9.77	14.73	11.35	12.63	10.94	Stress-activated kinase that phosphorylates c-Jun/AP-1, promoting expression of proinflammatory cytokines.	[50–52]
E9Q6I2	Il1rap	Interleukin 1 receptor accessory protein (IL-1RAcP)	10.03	12.49	9.80	9.71	11.95	Essential IL-1 family coreceptor; required for MyD88/IRAK recruitment and activation of NF-κB and AP-1.	[53]

Two negative regulators of cytokine signaling, interleukin-18-binding protein (IL-18BP) and suppressor of cytokine signaling 5 (SOCS5), were upregulated in IL15.3 treatment. The expression of IL-18BP shifted from a log_2_ value of 7.58 in the LPS control group to 8.45 at 100 µg/mL and 9.65 at 200 µg/mL IL15.3 (Table 4). IL-18BP is a soluble antagonist that binds IL-18 with high affinity, preventing IL-18 from engaging its receptor and activating downstream proinflammatory pathways [43,44]. By sequestering IL-18, IL-18BP limits IFN-γ induction and attenuates NF-κB signaling, both central to inflammatory amplification [43,44]. Therefore, the IL-18BP upregulation observed with IL15.3 treatment likely helps reduce the escalation of inflammation. Similarly, SOCS5 expression was enhanced following IL15.3 treatment, rising to a log_2_ value of 8.78 at 100 µg/mL and 8.45 at 200 µg/mL IL15.3. SOCS5, a member of the SOCS protein family, acts as a negative regulator of cytokine signaling by inhibiting the JAK/STAT pathway, which limits excessive inflammatory responses [45,46]. The induction of SOCS5 suggests that IL15.3 activates a broader spectrum of negative regulatory mechanisms to actively modulate the LPS-induced cellular response.

Five proteins associated with pro-inflammatory signaling exhibited reduced expression in IL15.3-treated groups compared to the LPS control, suggesting broad suppression across multiple cascades. Specifically, inhibitor of nuclear factor kappa-B kinase subunit beta (IKKβ), exhibited dramatic suppression, decreasing from a log_2_ expression level of 12.91 (LPS control) to 10.62, 10.15, and 7.85 at 50, 100, and 200 µg/mL IL15.3, respectively, representing a 33.54-fold reduction at the highest concentration (Table 4). IKKβ functions as the predominant catalytic subunit of the IKK complex that activates NF-κB signaling by phosphorylating IκBα, triggering its degradation and subsequent nuclear translocation of NF-κB to induce inflammatory gene expression [47]. Suppression of IKKβ expression suggests that IL15.3 may restrict the NF-κB signaling cascade at its initiation point, providing a fundamental anti-inflammatory mechanism.

Two members of the Janus kinase (JAK) family, which serve as essential mediators of cytokine receptor signaling, exhibited dose-dependent downregulation following IL15.3 treatment. Tyrosine-protein kinase JAK1 (JAK1) expression decreased from log_2_ 11.52 (LPS control) to 9.44, 7.66, and 9.96, while Tyrosine-protein kinase JAK3 (JAK3) declined from log_2_ 10.63 to 8.88, 7.81, and 7.31 at IL15.3 concentrations of 50, 100, and 200 µg/mL, respectively (Table 4). Both JAK1 and JAK3 are central mediators of cytokine-induced JAK–STAT signaling and thereby regulate inflammatory responses [6,48]. JAK1 transduces signals from pro-inflammatory cytokines, notably IL-6 and type I/II interferons (IFN), by phosphorylating receptor-associated STATs to drive transcription of inflammatory and acute-phase genes [6,48]. Selective JAK1 inhibitors reduced disease activity and inflammatory biomarkers in clinical trials for rheumatoid arthritis and atopic dermatitis, supporting JAK1 blockade as an effective anti-inflammatory strategy [6,48]. JAK3 is predominantly expressed in hematopoietic cells and mediates cytokine signaling for lymphocyte function and homeostasis [6]. JAK3 inhibition reduces inflammation in preclinical arthritis models and limits T cell activation and inflammatory gene expression [48,49].

IL15.3 treatment also resulted in dose-dependent suppression of two additional inflammatory mediators. Stress-activated protein kinase JNK (EC 2.7.11.24) decreased from a log_2_ expression level of 14.73 (LPS control) to 11.35, 12.63, and 10.94 at increasing doses. Interleukin-1 receptor accessory protein (IL-1RAcP) was similarly suppressed from 12.49 (LPS control) to 9.80, 9.71, and 11.95 across the treatment concentrations (Table 4). JNKs were first described as stress‑activated protein kinases because they are activated by a variety of environmental stresses and pro-inflammatory signaling. They were later called c‑Jun N‑terminal kinases to highlight their ability to phosphorylate the transcription factor c‑Jun [50,51]. Its inflammatory function involves phosphorylating c-Jun, leading to the formation of the Activator Protein-1 (AP-1) transcription factor, which regulates numerous pro-inflammatory genes [51]. Given the fundamental role of JNK in chronic inflammatory diseases [52], the dose-dependent suppression of JNK expression by IL15.3 suggests interference with a core inflammatory signaling cascade and highlights its potential as a mechanism-based response. IL-1RAcP serves as an essential co-receptor for IL-1 family cytokines (IL-1, IL-33, IL-36), enabling receptor complex formation and recruitment of signaling mediators MyD88 and IRAK, which activate NF-κB and AP-1 transcription factors [53]. Dysregulated IL-1RAcP signaling contributes to chronic inflammatory disorders, establishing it as a potential therapeutic target [53].

Overall, the coordinated upregulation of negative regulators (e.g., IL-18BP, SOCS5) together with downregulation of central pro-inflammatory mediators (e.g., IKKβ, JAK1/3, JNK, IL-1RAcP) suggests that IL15.3 may attenuate core inflammatory pathways in LPS-stimulated macrophages.

### 3.5. Effects of IL15.3 on the expression of inflammation- and antioxidant-related genes RAW 264.7 cells

To elucidate the anti-inflammatory mechanism of IL15.3, its effect on the expression of key pro-inflammatory genes was evaluated in LPS-stimulated RAW 264.7 macrophages using quantitative real-time PCR (qRT-PCR). Upon exposure to LPS, TLR4 on RAW 264.7 macrophages is activated, initiating signaling cascades that involve NF-κB and MAPK pathways. These pathways promote the nuclear translocation of transcription factors, ultimately inducing high levels of pro-inflammatory mediators such as inducible nitric oxide synthase (iNOS) (which produces nitric oxide, NO), cyclooxygenase-2 (COX-2), tumor necrosis factor-alpha (TNF-α), and interleukin-6 (IL-6) [32]. This coordinated response is fundamental to the macrophage-driven amplification of inflammation following bacterial endotoxin exposure. Consistent with this mechanism, LPS treatment significantly increased the mRNA expression of *iNOS*, *COX-2*, *TNF-α*, and *IL-6* compared to untreated controls (Fig 6). Such elevations are established markers of classical inflammatory activation in this model [54]. The robust upregulation of these targets demonstrates the reliability of the LPS-RAW 264.7 cell system for investigating anti-inflammatory agents.

**Fig 6:**
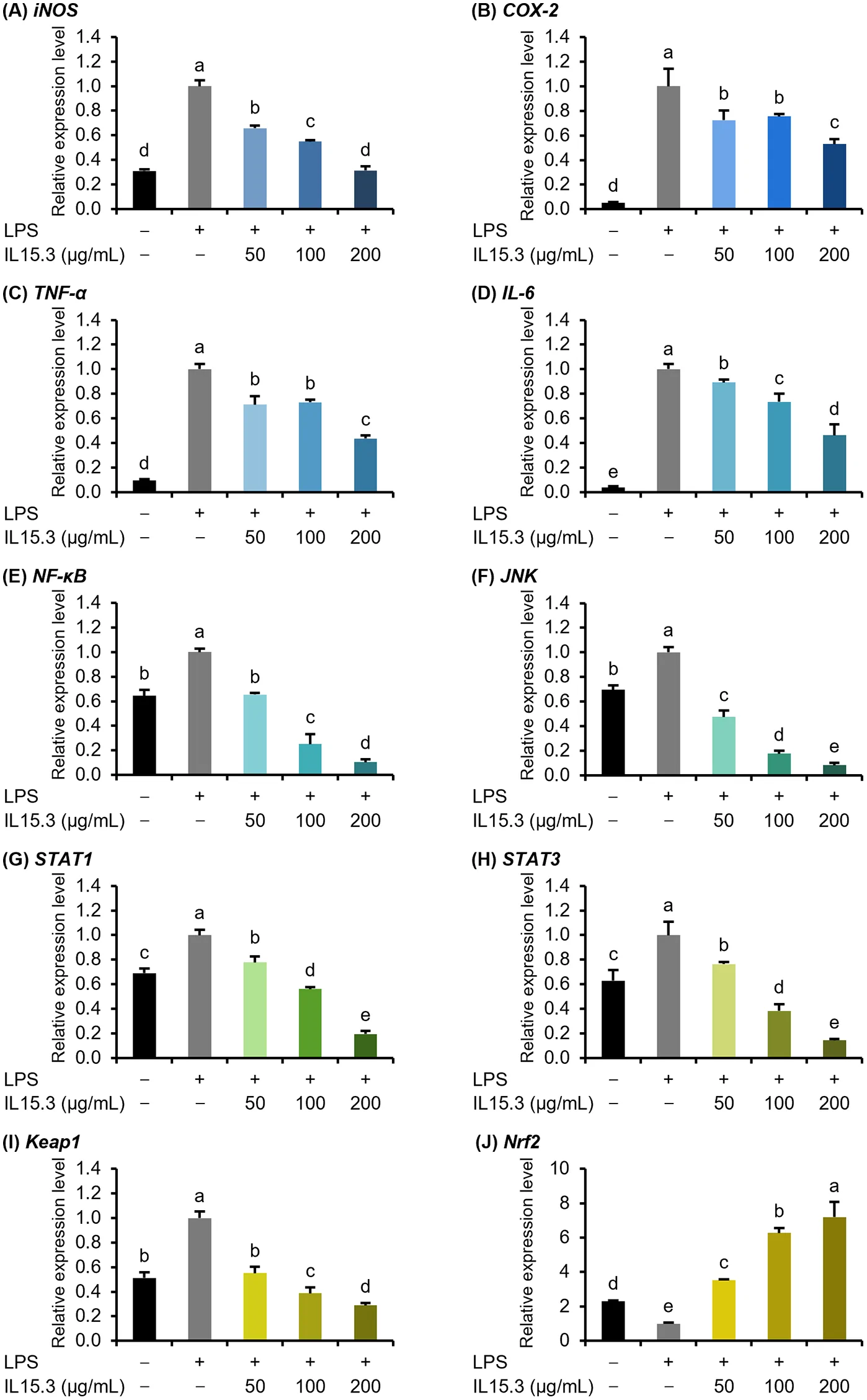
Transcriptional modulation of inflammatory mediators, signaling pathways, and antioxidant response by IL15.3 in LPS-stimulated RAW 264.7 macrophages. Relative mRNA expression levels of (A) *iNOS*, (B) *COX-2*, (C) *TNF-α*, (D) *IL-6*, (E) *NF-κB*, (F) *JNK*, (G) *STAT1*, (H) *STAT3*, (I) *Keap1*, and (J) *Nrf2* were analyzed by qRT-PCR following treatment with 100 ng/mL LPS in the presence or absence of IL15.3. Gene expression was normalized to β-actin and presented as fold-change relative to the LPS-treated control group (set as 1.0). Data are presented as mean ± standard deviation from three independent experiments. Statistical analysis was performed using one-way ANOVA followed by Duncan’s multiple range test; different letters indicate significant differences (*p* < 0.05).

Crucially, co-treatment with IL15.3 (50–200 µg/mL) significantly suppressed this LPS-induced expression across all evaluated genes: *iNOS* (Fig 6A), *COX-2* (Fig 6B), *TNF-α* (Fig 6C), and *IL-6* (Fig 6D). These transcriptional data provide a molecular basis for the previously observed peptide-mediated inhibition of NO production (Fig 2). Specifically, a clear dose-dependent reduction was observed for *iNOS* and *IL-6*, with each tested concentration producing a significantly greater reduction than the preceding one. In contrast, while *COX-2* and *TNF-α* mRNA levels were significantly reduced relative to the LPS control across all tested concentrations, they exhibited comparable suppression between 50 and 100 µg/mL, followed by a further significant decrease at 200 µg/mL. Collectively, these transcriptional data demonstrate that IL15.3 suppresses key pro-inflammatory mediators at the transcriptional level, with the greatest suppression of all four genes observed at 200 µg/mL.

To further examine the mechanisms suggested by the proteomic profile, we assessed expression of representative NF-κB- and JAK/STAT-associated transcripts. *NF-κB* (Fig 6E) and *JNK* (Fig 6F) mRNA levels were significantly downregulated in LPS + IL15.3-treated cells relative to LPS alone, a pattern consistent with proteomic data showing increased NF-κB-suppressive proteins (ISG15 [33,34], Tnip3 [37,38], PR [39], pro-neuregulin-1 [40], Dab2ip [41,42], IL-18BP [43,44]) and decreased NF-κB-activating proteins (IKKβ [47], IL-1RAcP [53]). This pattern is also consistent with reduced JNK expression, given its role in promoting pro-inflammatory cytokine production [50–52]. Similarly, *STAT1* (Fig 6G) and *STAT3* (Fig 6H) expression were reduced, consistent with the proteomic finding of increased SOCS5, a reported negative regulator of JAK/STAT signaling [45,46], and with the known role of JAK1/3 in activating STAT-mediated inflammatory transcription [6,48,49].

Given the observed suppression of ROS production, we further examined the Keap1/Nrf2 antioxidant pathway. LPS + IL15.3 co-treatment significantly downregulated *Keap1* (Fig 6I) and upregulated *Nrf2* (Fig 6J) mRNA relative to LPS alone. As Keap1 normally represses Nrf2 by promoting its cytoplasmic degradation [55], this reciprocal pattern suggests that IL15.3 may relieve Keap1-mediated repression, favoring Nrf2-dependent antioxidant responses, and is consistent with the proteomic increase in PEX14, a ROS-neutralizing peroxisomal protein [35,36].

Taken together, these transcriptional data provide supportive evidence that aligns with our proteomic findings, suggesting that IL15.3 may help alleviate LPS-induced inflammatory responses in RAW 264.7 macrophages partly through pathways related to NF-κB, JAK/STAT, and Keap1/Nrf2 signaling.

### 3.6. Molecular docking analysis suggests potential interactions of IL15.3 with iNOS and the Keap1 Kelch domain

The experimental data showed that IL15.3 reduced LPS-induced NO production and iNOS expression, as well as intracellular ROS accumulation, in RAW 264.7 macrophages. To probe the structural basis of these two phenotypes, molecular docking was performed against iNOS, which directly drives inflammatory NO production [56], and against the Kelch domain of Keap1, a key regulator of the cellular antioxidant response through its interaction with Nrf2 [57]. This computational approach was used to examine whether IL15.3 could plausibly engage these two targets at the protein level, complementing the transcriptional and biochemical findings.

Docking against iNOS predicted a favorable interaction, with a binding free energy of −33.39 kcal/mol (S1 Table). IL15.3 was positioned within a surface pocket of iNOS (Fig 7A) and stabilized predominantly through hydrophobic contacts, together with several hydrogen bonds (Fig 7B). Hydrophobic interactions were predicted with K117, T120, Y485, Q381, I271, A275, L278, E488, W457, and A276, while hydrogen bonds involved T120, Q265, and E297, with bond distances ranging from 2.39 to 2.95 Å (S1 Table). Because these contacts are distributed across the binding interface rather than concentrated at a single polar site, IL15.3 appears able to adopt a relatively well-defined binding orientation on iNOS, consistent with a stable predicted complex. This structural picture aligns with the concentration-dependent reduction in NO production observed in LPS-stimulated macrophages, suggesting that beyond suppressing iNOS at the transcriptional level, IL15.3 may also associate directly with the iNOS protein.

**Fig 7:**
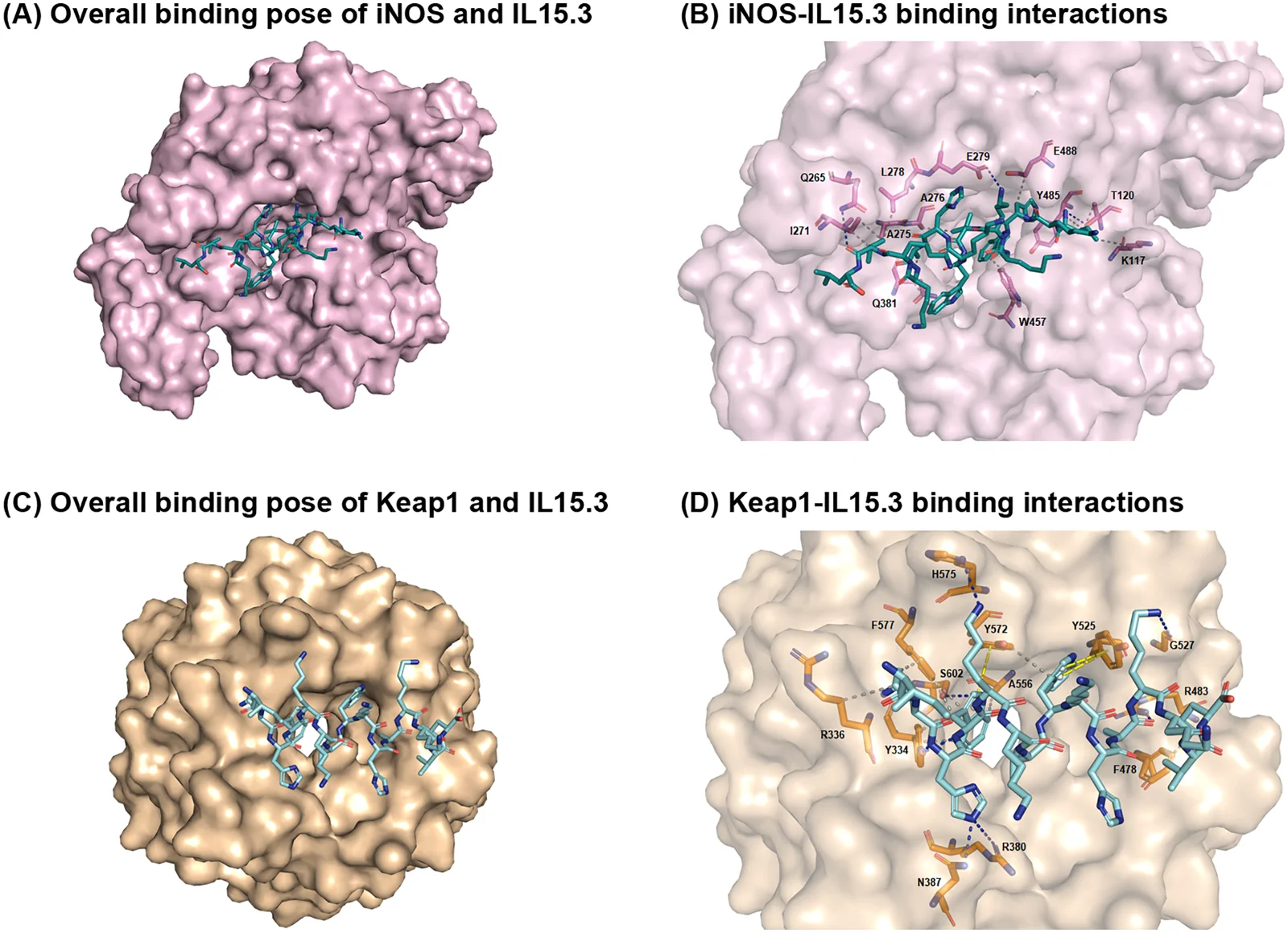
Peptide–protein docking complexes of IL15.3 against iNOS and the Kelch domain of Keap1. Overall binding poses of IL15.3 against iNOS (A) and Keap1 (C). Close-up views of the corresponding binding interactions for iNOS (B) and Keap1 (D). iNOS is colored pink, Keap1 is colored orange, and IL15.3 is colored cyan in all panels. Dashed lines indicate hydrogen bonds (blue), hydrophobic interactions (grey), and π-stacking interactions (yellow).

Relative to iNOS, the predicted interaction between IL15.3 and the Keap1 Kelch domain was even more favorable, with a binding free energy of −40.49 kcal/mol (S1 Table; Fig 7C). This complex was stabilized by a more extensive network of interactions, including hydrogen bonds (R483, G527, Y334, R380, N387, S602, and H575), hydrophobic contacts (F478, R483, R336, F577, Y334, A556, Y525, and Y572), and π-stacking interactions (Y572 and Y525). Notably, several residues — Y334, F577, Y525, and Y572 — participated in more than one type of interaction (S1 Table; Fig 7D), indicating that these aromatic residues likely make a substantial contribution to stabilizing IL15.3 within the Keap1 binding region. The greater number and diversity of contacts compared with iNOS is consistent with the more favorable binding energy calculated for this complex, and is particularly relevant given that the Kelch domain mediates recognition of regulatory partners such as Nrf2 [58]. Peptide binding within this region could therefore plausibly interfere with Keap1-mediated regulation of Nrf2, offering a structural explanation for the progressive reduction in intracellular ROS observed with IL15.3 treatment.

Together, these docking results link the two experimentally observed phenotypes to distinct but complementary structural hypotheses: the predicted interaction with iNOS is consistent with reduced NO production and iNOS transcription, while the stronger predicted interaction with the Keap1 Kelch domain offers a plausible basis for the reduction in intracellular ROS. Taken together with the experimental data, these findings support a multimodal mechanism in which IL15.3 attenuates inflammatory signaling and oxidative stress through coordinated effects at the transcriptional, proteomic, and potentially protein-interaction levels.

## 4. Conclusions

This study demonstrates that the antimicrobial peptide IL15.3 exerts anti-inflammatory and antioxidant activity in LPS-stimulated macrophages while exhibiting low host cytotoxicity. IL15.3 suppresses key proinflammatory mediators (NO, *iNOS*, *COX-2*, *TNF-α*, *IL-6*) and modulates multiple signaling nodes—downregulating central effectors of NF-κB, JNK, and JAK/STAT signaling (e.g., IKKβ, JAK1/3, STAT1/3) and upregulating protective regulators (e.g., IL-18BP, SOCS5, ISG15). IL15.3 further attenuates intracellular ROS accumulation, associated with reciprocal regulation of the Keap1/Nrf2 antioxidant pathway. *In silico* molecular docking analysis predicts potential interactions of IL15.3 with iNOS and the Keap1 Kelch domain, suggesting structural insight consistent with the observed transcriptional and proteomic effects. A schematic summary of the LPS-TLR4–mediated inflammatory cascade and the corresponding sites of IL15.3-mediated modulation is presented in Fig 8. Together, these transcriptional and proteomic data indicate that IL15.3 acts via a multi-target mechanism to attenuate macrophage activation and oxidative stress, restoring immune balance, supporting further mechanistic and *in vivo* evaluation of IL15.3 as a candidate for anti-inflammatory therapeutic.

**Fig 8:**
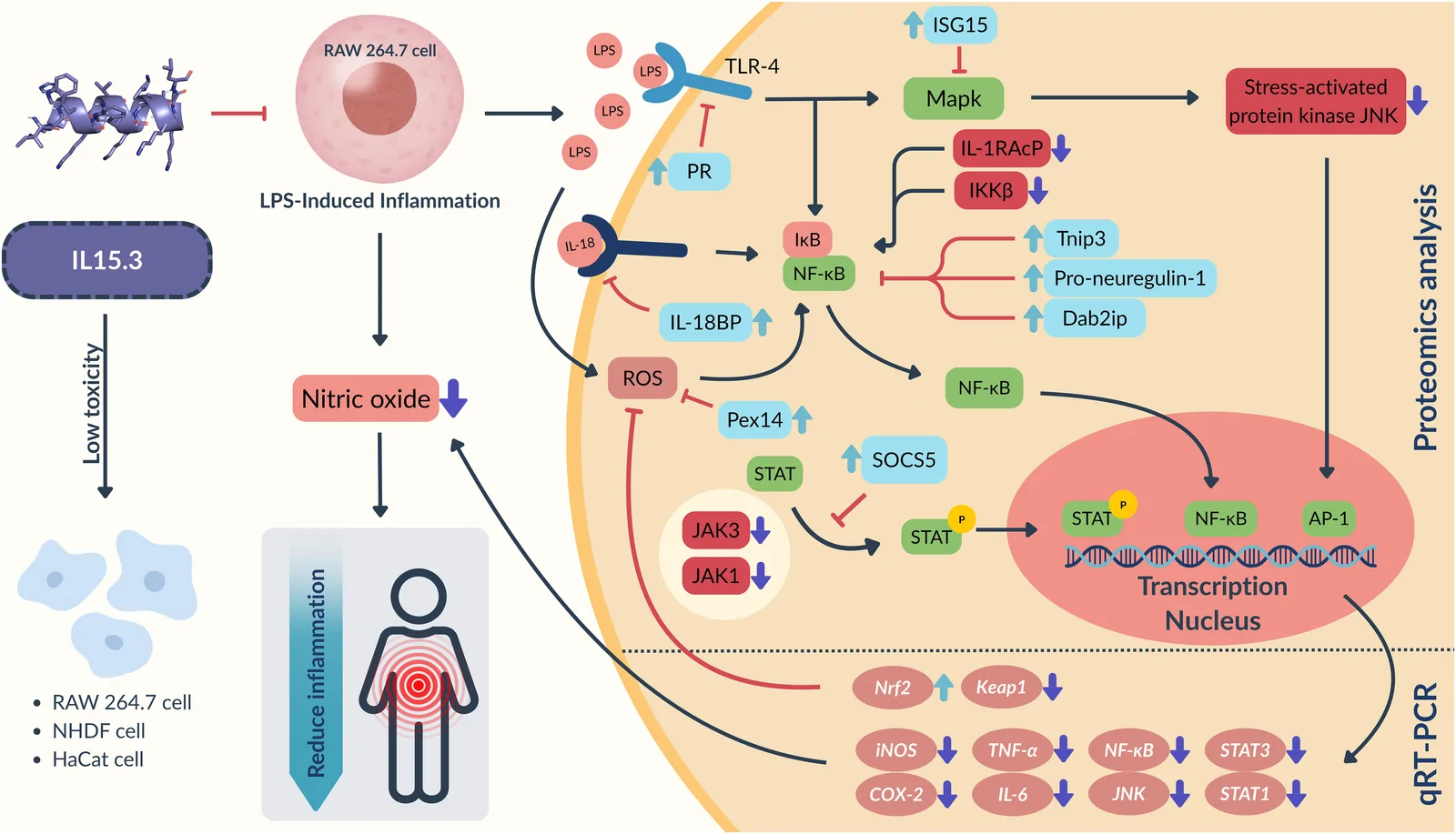
Schematic summary integrating transcriptional (qRT-PCR) and proteomic profiling of IL15.3-mediated anti-inflammatory and antioxidant mechanisms in LPS-stimulated RAW 264.7 macrophages. IL15.3 attenuates LPS/TLR4-induced inflammation and oxidative stress via multi-nodal regulation: (i) suppressing intracellular ROS accumulation and NO release by downregulating key pro-inflammatory and signaling genes (*iNOS*, *COX-2*, *TNF-α*, *IL-6*, *NF-κB*, *JNK*, *STAT1*, and *STAT3*); (ii) modulating redox balance through transcriptional regulation of *Keap1* and *Nrf2*; (iii) downregulating central proteomic effectors (e.g., IKKβ, JAK1/JAK3); and (iv) upregulating endogenous protective proteins (IL-18BP, SOCS5, ISG15). Red inhibitory bars (⊣) and purple downward arrows (↓) indicate downregulation/suppression, while cyan upward arrows (↑) indicate upregulation mediated by IL15.3. Black arrows denote signal activation.

## Supporting information

S1 Fig Structural characteristics and amphipathic α-helical projection of peptide IL15.3. (A) Helical wheel projection of IL15.3 (IKHWKKVWKHAKKVL) generated using the HeliQuest server, illustrating the facial amphipathicity with positively charged residues (Lys) segregated on the polar face and hydrophobic residues (Ile, Trp, Val, Ala, Leu) clustered on the opposing non-polar face. The arrow indicates the hydrophobic moment vector. (B) Predicted secondary structure model displaying an α-helical ribbon conformation, predicted using the PEP-FOLD server and rendered in PyMOL.
(PDF)

S1 Table Molecular docking analysis of IL15.3 against iNOS and Keap1. Binding free energies and key interacting residues between IL15.3 and the active sites of iNOS and Keap1.
(PDF)
