# Supplementary material for: IL15.3, a synthetic antimicrobial peptide, attenuates LPS-induced inflammatory responses in RAW 264.7 macrophages via transcriptional and proteomic modulation
Source: PLoS One. 2026 Oct 22;21(10):e0361031. doi: 10.1371/journal.pone.0361031 (PMC13690211; doi:10.1371/journal.pone.0361031)
Supplement: Supplementary file 1 [file pone.0361031.s001.pdf]

Supporting information

IL15.3, a synthetic antimicrobial peptide, attenuates LPS-induced inflammatory responses in RAW 264.7 macrophages via transcriptional and proteomic modulation

Chanapat Boonmark[1], Sittiruk Roytrakul[2], Fasawanya Saengyotha[1], Anupong Tankrathok[1,3], Anuwat Wanthong[1,4], Jureerut Daduang[5], Sakda Daduang[1,6], Arunrat Chaveerach[7], and Nisachon Jangpromma[1,8,*]

[1] Protein and Bio-Innovation Research Center for Sustainable Health (ProBISH), Faculty of Science, Khon Kaen University, Khon Kaen 40002, Thailand

[2] National Center for Genetic Engineering and Biotechnology, National Science and Technology Development Agency, Pathumthani, 12120, Thailand

[3] Faculty of Agricultural Technology, Department of Biotechnology, Kalasin University, 46000 Kalasin, Thailand

[4] Department of Biology, Faculty of Science, Mahasarakham University, Mahasarakham, 44150, Thailand

[5] Department of Clinical Chemistry, Faculty of Associated Medical Sciences, Khon Kaen University, Khon Kaen 40002, Thailand

[6] Department of Pharmacognosy and Toxicology, Faculty of Pharmaceutical Sciences, Khon Kaen University, Khon Kaen 40002, Thailand

[7] Department of Biology, Faculty of Science, Khon Kaen University, Khon Kaen 40002, Thailand

[8] Department of Biochemistry, Faculty of Science, Khon Kaen University, Khon Kaen 40002, Thailand

* Corresponding author. Department of Biochemistry, Faculty of Science, Khon Kaen University, Khon Kaen 40002, Thailand

E-mail address: nisaja@kku.ac.th (NJ).

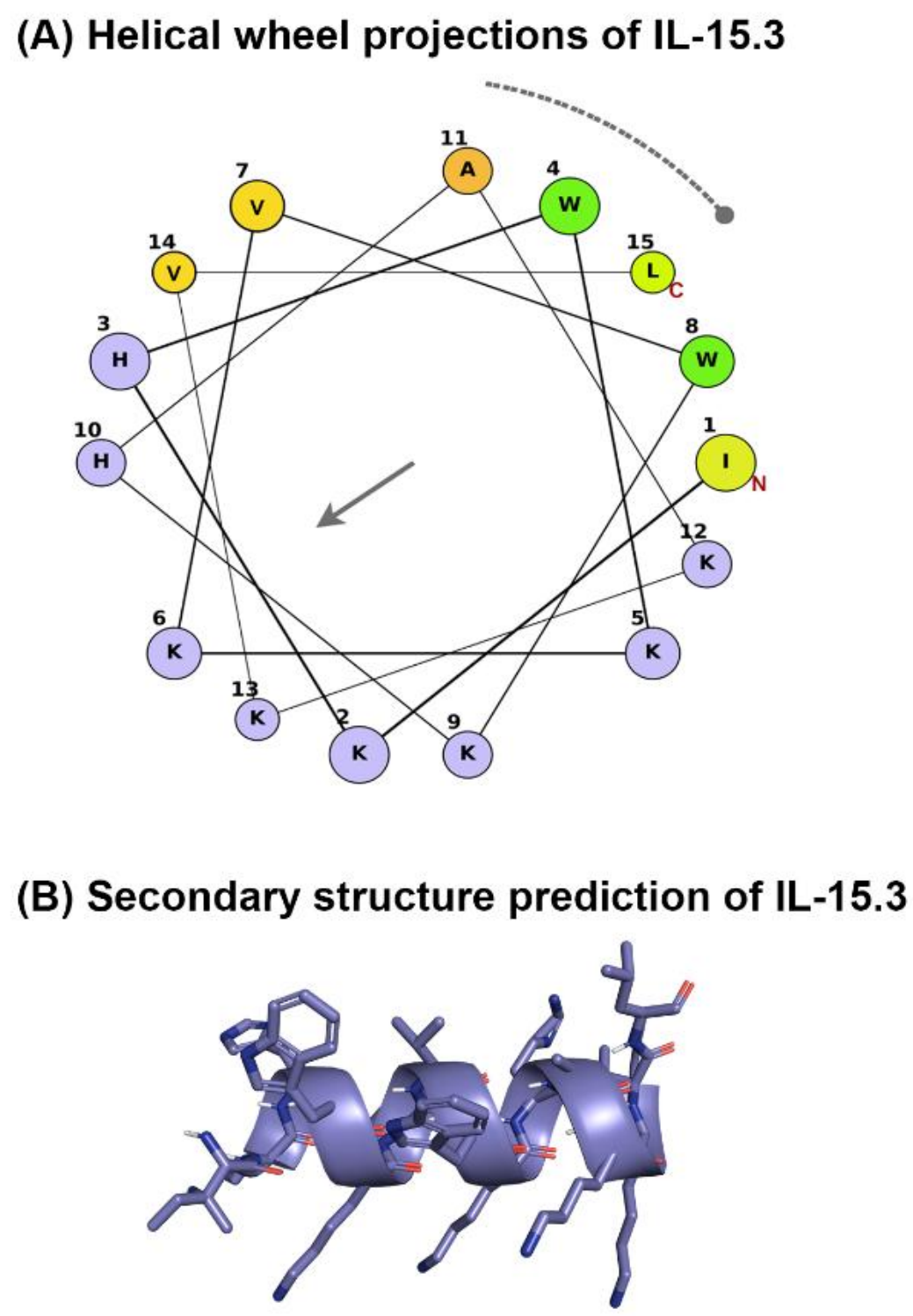

S1 Fig. Structural characteristics and amphipathic α-helical projection of peptide IL15.3. (A) Helical wheel projection of IL15.3 (IKHWKKVWKHAKKVL) generated using the HeliQuest server, illustrating the facial amphipathicity with positively charged residues (Lys) segregated on the polar face and hydrophobic residues (Ile, Trp, Val, Ala, Leu) clustered on the opposing non-polar face. The arrow indicates the hydrophobic moment vector. (B) Predicted secondary structure model displaying an α-helical ribbon conformation, predicted using the PEP-FOLD server and rendered in PyMOL.
