# Supplementary material for: IL15.3, a synthetic antimicrobial peptide, attenuates LPS-induced inflammatory responses in RAW 264.7 macrophages via transcriptional and proteomic modulation
Source: PLoS One. 2026 Oct 22;21(10):e0361031. doi: 10.1371/journal.pone.0361031 (PMC13690211; doi:10.1371/journal.pone.0361031)
Supplement: Supplementary file 2 [file pone.0361031.s002.pdf]

Supporting information

IL15.3, a synthetic antimicrobial peptide, attenuates LPS-induced inflammatory responses in RAW 264.7 macrophages via transcriptional and proteomic modulation

Chanapat Boonmark[1], Sittiruk Roytrakul[2], Fasawanya Saengyotha[1], Anupong Tankrathok[1,3], Anuwat Wanthong[1,4], Jureerut Daduang[5], Sakda Daduang[1,6], Arunrat Chaveerach[7], and Nisachon Jangpromma[1,8,*]

[1] Protein and Bio-Innovation Research Center for Sustainable Health (ProBISH), Faculty of Science, Khon Kaen University, Khon Kaen 40002, Thailand

[2] National Center for Genetic Engineering and Biotechnology, National Science and Technology Development Agency, Pathumthani, 12120, Thailand

[3] Faculty of Agricultural Technology, Department of Biotechnology, Kalasin University, 46000 Kalasin, Thailand

[4] Department of Biology, Faculty of Science, Mahasarakham University, Mahasarakham, 44150, Thailand

[5] Department of Clinical Chemistry, Faculty of Associated Medical Sciences, Khon Kaen University, Khon Kaen 40002, Thailand

[6] Department of Pharmacognosy and Toxicology, Faculty of Pharmaceutical Sciences, Khon Kaen University, Khon Kaen 40002, Thailand

[7] Department of Biology, Faculty of Science, Khon Kaen University, Khon Kaen 40002, Thailand

[8] Department of Biochemistry, Faculty of Science, Khon Kaen University, Khon Kaen 40002, Thailand

* Corresponding author. Department of Biochemistry, Faculty of Science, Khon Kaen University, Khon Kaen 40002, Thailand

E-mail address: nisaja@kku.ac.th (NJ).

S1 Table. Molecular docking analysis of IL15.3 against iNOS and Keap1. Binding free energies and key interacting residues between IL15.3 and the active sites of iNOS and Keap1.

Protein	Binding free energy of complex (kcal/mol)	Residue	Distance H-A	Interaction type
iNOS	-33.39	K117	3.40	Hydrophobic Interaction
		T120	2.95	Hydrophobic Interaction
		Y485	3.81	Hydrophobic Interaction
		T120	2.73	Hydrogen Bond
		Q381	3.22	Hydrophobic Interaction
		I271	2.55	Hydrophobic Interaction
		I271	3.27	Hydrophobic Interaction
		A275	3.67	Hydrophobic Interaction
		L278	3.18	Hydrophobic Interaction
		Q265	2.95	Hydrogen Bond
		E488	3.39	Hydrophobic Interaction
		W457	3.51	Hydrophobic Interaction
		E297	2.39	Hydrogen Bond
		A276	3.61	Hydrophobic Interaction
		Q381	3.97	Hydrophobic Interaction
Keap1	-40.49	R483	2.21	Hydrogen Bond
		G527	2.82	Hydrogen Bond
		F478	2.72	Hydrophobic Interaction
		R483	3.83	Hydrophobic Interaction
		R336	3.97	Hydrophobic Interaction
		F577	2.67	Hydrophobic Interaction
		Y334	2.43	Hydrogen Bond
		R380	3.35	Hydrogen Bond
		N387	2.77	Hydrogen Bond
		Y334	3.26	Hydrophobic Interaction
		Y334	3.49	Hydrophobic Interaction
		Y334	2.59	Hydrophobic Interaction
		A556	3.87	Hydrophobic Interaction
		F577	3.32	Hydrophobic Interaction
		F577	3.33	Hydrophobic Interaction
		Y334	1.88	Hydrogen Bond

Protein	Binding free energy of complex (kcal/mol)	Residue	Distance H-A	Interaction type
		S602	2.83	Hydrogen Bond
		Y572	4.67	π-Stacking
		H575	2.66	Hydrogen Bond
		Y525	2.98	Hydrophobic Interaction
		Y572	3.62	Hydrophobic Interaction
		Y525	4.01	π-Stacking
		Y525	4.25	π-Stacking
